# Applications of Osteoimmunomodulation Models in Evaluating Osteogenic Biomaterials

**DOI:** 10.3390/jfb16060217

**Published:** 2025-06-11

**Authors:** Yuhan Wang, Yuzhu He, Yaran Zang, Zijiao Zhang, Guangyao Li, Wenqi Fu, Guowu Ma

**Affiliations:** 1 School of Stomatology, Dalian Medical University, Lvshun South Road, Dalian 116044, China; 13604258520@163.com (Y.W.); yuzhuh0723@dmu.edu.cn (Y.H.); zangyaran0327@dmu.edu.cn (Y.Z.); zhangzj01@dmu.edu.cn (Z.Z.); 2 Harbin Institute of Technology Zhengzhou Research Institute, No. 26, Longyuan East Seventh Street, Zhengdong New District, Zhengzhou 450011, China; liguangyao@hitzri.cn; 3 Academician Laboratory of Immune and Oral Development & Regeneration, Dalian Medical University, Lvshun South Road, Dalian 116044, China

**Keywords:** biomaterials, bone regeneration, osteoimmunomodulation, experimental model

## Abstract

The development of osteogenic biomaterials relies on updates in research methodologies. Establishing reasonable modes is the basis for obtaining reliable experimental conclusions. With the advancement in bone immunology, osteoimmunomodulatory properties have become one of the crucial indexes for evaluating osteogenic biomaterials. Summarizing the current models of bone immunomodulation is beneficial for optimizing experimental protocols and promoting the clinical application of osteogenic biomaterials. In this review, we introduced the crosstalk between the skeletal system and the immune system, in particular, the roles of different immune cells in the process of bone regeneration. Moreover, the mechanisms of osteogenic biomaterials in regulating the osteoimmune microenvironment were analyzed, followed by summarizing the benefits and limitations of current osteoimmunomodulation models in evaluating osteogenic biomaterials. Finally, we discussed the expected future directions of the applications of osteoimmunomodulation models.

## 1. Introduction

Bone defects resulting from trauma, tumors, and congenital developmental diseases have a profound impact on the function of the patient’s skeletal system and quality of life [1]. Although autogenous bone represents the gold standard for bone defect repair, its clinical application is constrained by several factors [2]. These include donor site morbidity, limited graft quantity, post-graft resorption, prolonged healing time, risk of infection, and other complications [3]. The implantation of osteogenic biomaterials continues to be one of the common treatments [4]. Developing new biomaterials with efficient osteogenic efficacy depends on effective preclinical testing. Thus, the construction of a reasonable evaluation model is of significant importance for the development of osteogenic materials and the reduction in failure rates in clinical applications. In vivo, implanted osteogenic biomaterials, as foreign objects, can initiate immune responses and form a new microenvironment, which decides the fate of bone healing [5]. The term “osteoimmunology” was first proposed in 2000, marking the beginning of a new era in the study of the intricate relationships between the skeletal system and the immune system at the cellular and biomolecular levels [6,7]. This research field has led to significant advancements in evaluating osteogenic biomaterials [8]. After implanting osteogenic biomaterials, immune cells will be activated immediately, which crosstalk with bone-related cells to decide the implant fate. On the other hand, biomaterials modulate the phenotype and function of immune cells by releasing biophysical, biochemical, and biological cues. To investigate these complex interaction mechanisms, we further analyzed the establishment and applications of current osteoimmunomodulation models in evaluating osteogenic biomaterials. Finally, continuing challenges and future directions of the model development were discussed.

## 2. Crosstalk Between Bone Cells and Immune Cells in the Process of Bone Regeneration

**Figure 1:**
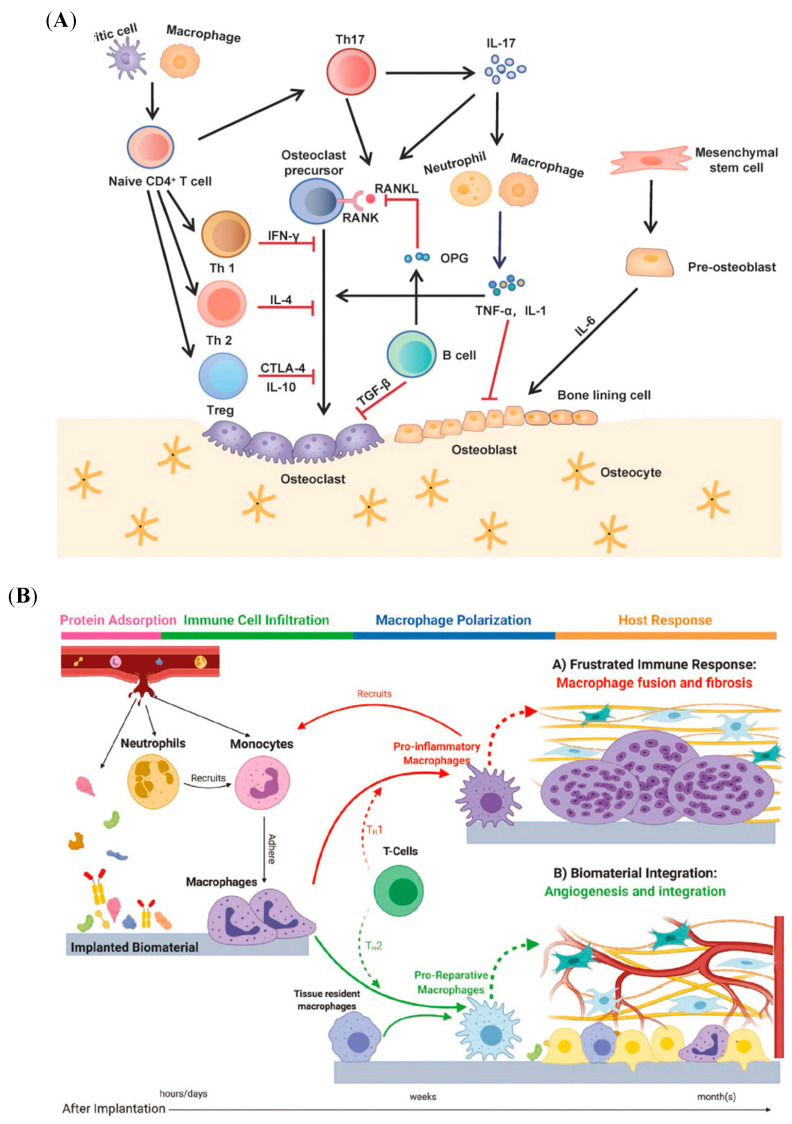
Schematic illustration of the crosstalk between bone and immune cells in biomaterial-induced bone regeneration. (**A**) The communication between bone-related cells and immune cells during bone formation (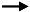 means promotion; 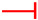 means inhibition). Reprinted from Ref. [11]. (**B**) The local immune–bone–biomaterial microenvironment. Reprinted with permission from Ref. [14]. Copyright 2020, John Wiley and Sons.

Bone regeneration is a dynamic, complex, and orderly biological process that usually undergoes three overlapping phases: inflammation, bone formation, and remodeling [9]. In these processes, osteoblasts (OBs) continuously receive biological signals from the surrounding environment. The modern theory of “osteoimmunology” indicates that bone regeneration results from the crosstalk of skeletal and immune systems (Figure 1A) [10,11]. Immune cells, as the main players in the inflammatory response, are involved in establishing the immune microenvironment for bone regeneration [12]. On the other hand, bone cells, especially bone marrow mesenchymal stem cells (BMSCs), can regulate immune cell polarization and behavior [13].

Osteogenic biomaterials implanted as foreign bodies can stimulate a series of host responses, including protein adsorption, immune cell infiltration, macrophage polarization, and host response, as shown in Figure 1B [14]. Initial protein adsorption affects immune cell recruitment and adhesion, followed by immune cell polarization [14,15,16]. Immune cells with different phenotypes influence the bone development microenvironment by releasing receptor activators of nuclear factor κ-B ligand (RANKL), interleukin-1β (IL-1β), interleukin-6 (IL-6), interleukin-10 (IL-10), and tumor necrosis factor α (TNF-α), which participate in deciding the fate of bone formation [17]. In turn, bone cells recruit immune cells by secreting chemokines, such as macrophage chemoattractant protein-1 (MCP-1) and stromal cell-derived factor 1 (SDF-1). This close relationship between the skeletal system and the immune system claims that changes in the physiology and pathology of one system can impact the other.

### 2.1. The Roles of Neutrophils in Bone Regeneration

Neutrophils are the most abundant type of leukocyte derived from hematopoietic stem cells in the bone marrow, constituting about 50–70% of the white blood cells, particularly during the foreign body response (FBR) [18,19]. After implanting, osteogenic biomaterials are recognized as foreign and activate the immune systems [20]. Neutrophils are among the first immune cells to be recruited in the implant site in response to danger-associated molecular patterns (DAMPs) released by damaged tissues, such as interleukin-8 (IL-8), leukotriene B4 (LTB4), and reactive oxygen species (ROS) [21]. These recruited neutrophils will also recruit further immune cells by secreting IL-8, macrophage inflammatory protein-1 (MIP-1), MCP-1, and matrix metalloproteinase-9 (MMP-9) [22]. At this initial stage, neutrophils help to clear debris and pathogens from the injury site, which is the process of engulfing and digesting foreign particles or damaged cells produced by implantation surgery. Besides discussing the spatiotemporal dynamics of neutrophils, advanced studies focused on the phenotypes in bone regeneration [23]. Zvi G Fridlender et al. first suggested that neutrophils could be polarized to “N1” or “N2” phenotypes, like M1 and M2 in macrophages, by establishing a flank tumor model in mice [24]. And then, neutrophils are classified into inflammatory N1 and regenerative N2 phenotypes [25]. A recent study indicated that neutrophils played an essential role in the initial stage of bone regeneration [23]. Furthermore, in the mice model, N2-polarized neutrophils recruited BMSCs by secreting stromal cell-derived factor-1α (SDF-1α), followed by increased migration of BMSCs by activating the phosphoinositide 3-kinase (PI3K)/protein kinase B (Akt)/*β*-catenin pathway [23].

Extracellular vesicles (EVs) contain rich biological information, which can be released by various cells, including neutrophils. Le Wang et al. pretreated BMSCs with neutrophil-EVs to make neutrophil-EVs-integrated BMSC sheets (BS@PMN-EVs) in vitro, followed by implanting the BS@PMN-EVs in the cranial defect model of rats [26]. The results of this study indicated that neutrophil-EVs promoted BMSC proliferation and osteogenic differentiation; further, BS@PMN-EVs enhanced the new bone maturation in vivo (BV/TV revealed new bone formation at ∼39.5%, 5.8%, and 23.8% upon the implantation of BS@PMN-EVs, the blank, and BS@PBS) [26]. In addition, neutrophils are involved in regulating bone regeneration by interacting with other cell types. They contributed to reprogramming macrophages and T cells to M2 and Treg phenotypes, respectively, which benefit osteogenic differentiation [23,27]. In addition, the study by TDK Herath et al. suggested that neutrophils promote angiogenesis and osteogenesis, two critical and interrelated processes in bone formation [28]. In particular, they established a novel triple cell co-culture model, consisting of neutrophils, human umbilical vein endothelial cells (HUVECs), and human osteoblasts (HOBs) [28]. Further results showed that neutrophils promoted the expression of angiogenic markers in HUVECs, such as vascular endothelial growth factor A (VEGF-A), CD34, and fibroblast growth factor-2 (FGF-2), and it increased the levels of alkaline phosphatase (ALP), osteocalcin (OCN), type I collagen (COL-1), and osteopontin (OPN) in HOBs [28]. Taken together, neutrophils perform multifaceted roles in regulating bone regeneration by clearing debris, modulating the inflammatory response, and influencing the activity of bone-related tissue cells.

### 2.2. The Roles of Dendritic Cells (DCs) in Bone Regeneration

The DCs are acknowledged as professional antigen-presenting cells (APCs) while sharing certain common features with osteoclasts (OCs) [29]. Both of them originate from hematopoietic tissue and display phagocytic capabilities. In steady conditions, there were rarely DCs in the bone tissue and no absence of bone in DC-deficient mice, which suggested that DCs do not exert a major role in normal bone [30]. However, DCs can act as precursors to OCs under inflammation, which are involved in the bone damage process [31]. Rivollier et al. first proposed that immature DCs can transdifferentiate into functional OCs [32]. The DC-derived OCs are further directly involved in the development of inflammatory bone disease [32]. This process may contribute to the sensitivity of DCs to RANKL. Although RANKL was first described as an important regulator of interactions between T cells and DCs, studies have demonstrated that RANKL can also influence the behavior of DCs [33]. Further, RANKL can protect mature DCs and prolong their survival, followed by promoting the production of pro-inflammatory cytokines [34,35]. In addition to its role in immunomodulation, DCs were transdifferentiated into mature resorbing OCs by treatment with RANKL in an inflammation model [36]. Thereby, DCs are a critical pool of OCs under inflammatory conditions to directly regulate bone homeostasis. A single-cell sequencing (scRNA-seq) analysis of the patient’s program showed that more CD14^+^ DCs existed in nonunions compared to the native bone [37]. These DCs were proposed to differentiate into OC-like cells, which may induce bone healing failure due to the increase in bone resorption [38]. The above studies indicated that the roles of DCs in the bone system depend on the specific conditions. Furthermore, the mechanisms of DCs in bone regeneration should be analyzed in the optimal model based on the purpose of the studies.

### 2.3. The Roles of Lymphocytes in Bone Regeneration

The two primary types of lymphocytes are T cells and B cells. Both originate from hematopoietic stem cells in the bone marrow and play crucial roles in bone formation [39]. In the process of bone regeneration, T cell and B cell infiltration presented two waves, during days 3–7 and after day 14, respectively [40].

#### 2.3.1. T Cells

The T cells regulate bone regeneration by secreting cytokines and interacting with other cells. Based on the expression of surface markers and the cytokines they produce, T cells are mainly classified into helper T cells (Th, CD4^+^ T cells) and cytotoxic T cells (CD8^+^ T cells) [41]. CD4^+^ T cells can be polarized to Th1 and Th17 subsets, followed by secreting the inflammatory cytokines, such as interferon-γ (IFN-γ), IL-17, and TNF-α, to form a pro-inflammatory microenvironment and induce heavy macrophage infiltration [42]. On the other hand, the subsets of Th2 and Treg reduce tissue inflammation by secreting interleukin-4 (IL-4), IL-10, and transforming growth factor-β (TGF-β) [43]. To explore the effects of T cells on bone regeneration of titanium (Ti) biomaterial implantation, Derek Avery et al. established the model of CD4 and/or CD8 knockout mice, and T cells were extracted from the treated mice (WT, Cd4^−/−^, or Cd8^−/−^), respectively. These different groups of T cells were directly co-cultured with macrophages to investigate the impact of CD4^+^ and CD8^+^ T cells on macrophage polarization in vitro [44]. Further, the authors made the macrophage-T cell-MSC co-culture model by culturing adipose-derived MSCs in transwell or conditioned media (CM) of macrophage-T cell co-culture [44]. The in vitro results were finally confirmed by implanting Ti implants in WT, Cd4^−/−^, and Cd8^−/−^ mice [44]. The results of this study demonstrated that the deficiency of either CD4^+^ or CD8^+^ T cells increased the proportion of pro-inflammatory macrophages, diminished MSC recruitment, and reduced new bone formation at the implantation site [44]. Moreover, compared to CD8^+^ T cell deficiency, CD4^+^ T cell deficiency exacerbated these effects [44].

As mentioned above, CD4^+^ T cells were further divided into Th1, Th2, Treg, and Th17 subsets. Current evidence suggests that Th1 cells primarily produce IFN-γ and Th2 cells secrete IL-4 and interleukin-13 (IL-13), thereby participating in regulating the behaviors of OCs and OBs [45,46]. However, the detailed mechanisms remain to be explored. In addition, Treg cells produce anti-inflammatory cytokines such as IL-10 and TGF-β, which can suppress bone resorption by inhibiting the activation of osteoclast precursors and promoting osteoprotegerin (OPG) expression, a decoy receptor for RANKL [47]. In the last few years, Th17 cells have attracted wide attention. They mainly promote bone resorption by secreting pro-inflammatory cytokines, such as interleukin-17 (IL-17), interleukin-22 (IL-22), and interleukin-26 (IL-26) [48,49]. In particular, IL-17 stimulates the production of RANKL by OBs, thereby promoting osteoclast differentiation, followed by bone resorption [49]. Moreover, CD8^+^ T cells can inhibit osteoclastogenesis and bone resorption by releasing IFN-γ, similar to the effects mediated by Th1 cells [50]. However, the precise mechanisms by which CD8^+^ T cells regulate bone cells are still being elucidated. A recent study explored the role of MSCs-based therapy in bone defect healing by Cytometry by Time Of Flight (CyTOF), which revealed the cellular and immunomodulatory profiles [51]. The results showed that MSCs increased the T cell population to about 24%, but the control group varied from 1% to 40% [51]. Further, CD4^+^ T cells consisted of about 68% of all T cells in the MSCs group, whereas CD8^+^ T cells were about 10% [51]. However, double-negative T cells (about 86%) formed a major population in the control group [51]. Therefore, the modulation of T cell subtypes by designing osteogenic biomaterials, which in turn promotes osteogenesis, deserves further investigation.

#### 2.3.2. B Cells

B cells are mainly involved in the process of adaptive immune response [52]. Similar to T cells, the differentiation and maturation of B cells are also closely related to osteogenesis. The RANKL/RANK/OPG axis contributes to recruiting B cells and the crosstalk between B cells and OBs [33]. More than two decades ago, William C. Dougall et al. established the RANK-deficient mice (RANK^−/−^) [53]. Following this model, RANK^−/−^ mice exhibited obvious osteopetrosis as a consequence of the impeded differentiation of OCs [53]. At the same time, a deficiency of B cells was observed within the spleen [53]. However, RANK is not necessary for the development and function of macrophages and DCs [53]. In the same year, Young-Yun Kong et al. found that RANKL was a new regulator in the early differentiation of B cells and an essential factor in osteoclast differentiation by establishing the RANKL-deficient mice (RANKL^−/−^) [54]. In addition to being regulated by RANKL, B cells can also produce RANKL to maintain their development in normal conditions instead of keeping bone homeostasis [55]. To further examine the relationship between B cells and bone-related cells, some studies have concentrated on the impact of B cells in pathological conditions. In the ovariectomy (OVX) mouse model, precursors of B (pre-B) cells were selectively increased in bone marrow, and osteoclastogenesis was enhanced, which may be attributed to estrogen deficiency [56,57]. Moreover, a study indicated that RANKL overexpression in pre-B cells induced by estrogen deficiency may be a significant contributor to the acceleration of osteoclastogenesis [58]. Another study further demonstrated that this accelerated osteoclastogenesis resulted from the accumulation of osteoclast precursors, followed by abnormal bone resorption [59]. The authors then suggested that estrogen-deficiency-induced pre-B cells could differentiate into OCs by using in vitro co-culture models [59]. Interestingly, mature B cells were found to inhibit osteoclastogenesis and shorten the life span of OCs by secreting the apoptotic cytokine TGF-β [60]. The study of B Klausen et al. also showed that a temporary and moderate B cell deficiency could aggregate the periodontal bone loss in a rat model of periodontitis [61], suggesting that B cells may exert osteoprotective effects in some pathological conditions. The communication between B cells and bone cells depends on the developmental stage of the B cells and the surrounding conditions; thus, it is essential to establish rational models to explore their potential mechanisms.

### 2.4. The Roles of Macrophages in Bone Regeneration

Among these immune cells, macrophages are regarded as playing a central role in osteogenesis due to their high plasticity and pivotal role in regulating the osteogenic microenvironment [10]. Macrophages have a spectrum of activation states, ranging from pro-inflammatory M1 to anti-inflammatory M2. In the early phase of bone damage, macrophages polarize into M1 phenotype through “classical activated”, which clears cell debris through phagocytosis and secreting pro-inflammatory cytokines, such as IL-1β, IFN-γ, and TNF-α [62]. This clearance function was a critical process in creating a conducive environment for subsequent bone formation to maintain skeletal metabolism. In addition, M1 can recruit stem cells and initiate the bone healing process by secreting cytokines such as TNF-α, oncostatin M (OSM), bone morphogenetic protein 2 (BMP-2), and bone morphogenetic protein 6 (BMP-6) [63,64]. However, the prolonged presence of M1 is demonstrated to be associated with the failure of bone regeneration [65]. During the subsequent bone regeneration phase, macrophages transform into an “alternatively activated” M2 phenotype, which supports bone repair via secreting osteogenesis-related proteins BMP-2, VEGF, and TGF-β1 [66]. At the same time, an array of anti-inflammatory cytokines, including IL-4, IL-10, and IL-13, are also released to establish a microenvironment supportive of bone healing [67]. As mentioned above, both M1 and M2 participate in bone formation, and appropriate switching of M1/M2 is crucial for achieving success in bone regeneration.

Native bone is a highly vascularized tissue. Adequate blood supply is involved in the regeneration of bone tissue, to ensure the enough oxygen and nutrients are delivered to activate OBs [68]. Thus, the vascularized osteogenesis generated by osteogenic biomaterials plays a crucial role in remodeling the bone defect [69]. In addition to directly regulating bone-related cells, macrophages can regulate bone formation by promoting angiogenesis, serving as a bridge [70]. A topological scaffold was functionalized with M2 macrophage-derived exosome (M2-exosome) and measured for inflammatory regulation property and osteogenic potential in both in vitro and in vivo experiments [71]. This study indicated that, based on the scaffold biomaterial, M2-exosome played the coupling effect of angiogenesis, osteoclastogenesis, and osteogenesis by stimulating type H vessel formation [71]. In addition to directly promoting osteogenesis by regulating macrophage polarization, macrophage-induced angiogenesis can be used to guide osteogenic biomaterial design.

### 2.5. The Roles of BMSCs in Regulating Immune Cells

The BMSCs are directly involved in bone regeneration due to their multidirectional differentiation potential, and they have been used as seed cells to treat bone defects [1,72]. In addition, they can create specific microenvironments by releasing various cytokines, chemokines, and exosomes to communicate with other cells, including immune cells [73]. Dandan Chen et al. demonstrated that BMSCs exert immunosuppressive effects by regulating T cell survival and differentiation using a transwell model in vitro [74]. BMSCs and LPS-stimulated CD4^+^ T cells were seeded in the upper and lower parts of the transwell dishes, respectively. The results showed that BMSCs suppressed T cell proliferation and reduced Th1/Th2 and Th17/Treg ratios by secreting cytokines and decreasing programmed cell death-1 (PD-1) expression in T cells [74].

In addition to the major paracrine pathways, immune cells can interact with BMSCs through direct cell–cell contact [75]. When the combination of BMSCs and T cells was directly cocultured, PD-ligand 1 (PD-L1) shRNA-BMSCs might activate CD4^+^ T cells. The vivo mice model further confirmed that PD-L1 inhibition attenuated BMSCs-induced imbalance of Th1/Th2 and Th17/Treg. These results suggested that the BMSCs interact with T cells via the PD-1/PD-L1 pathway [74]. The immune and bone systems were closely linked with each other; however, the detailed mechanism between them remains to be further explored. To investigate bone formation mechanisms and develop bone regeneration biomaterials, it is necessary to establish rational in vitro and in vivo research models.

## 3. Mechanisms of Osteogenic Biomaterials in Regulating Osteo-Immune Microenvironment

In contrast to the previous focus on the relationships between osteogenic biomaterials and OBs, the profound influence of osteogenic biomaterials in the immune response-modulated osteogenesis attracts wide attention. Based on the tight relationship between bone and immune system, the development of osteogenic biomaterials evolved from only focusing on mechanical-physical-chemical principles and directly promoting osteoblastic lineage cells to regulating bone-immune microenvironment, which is called “osteoimmunomodulation” [76]. Macrophages are extensively researched due to their high plasticity and critical role in regulating the osteoimmune microenvironment. The relevant studies are listed in Table 1.

The macrophages sense and respond to the endogenous and exogenous stimuli in the rounding microenvironment. As mentioned above, the M1 and M2 phenotypes participate in different stages of bone formation. Thus, understanding the potential mechanisms of osteogenic biomaterials regulating macrophage polarization is crucial, as it could guide the development of the next biomaterials.

### 3.1. Biophysical Cues

**Figure 2:**
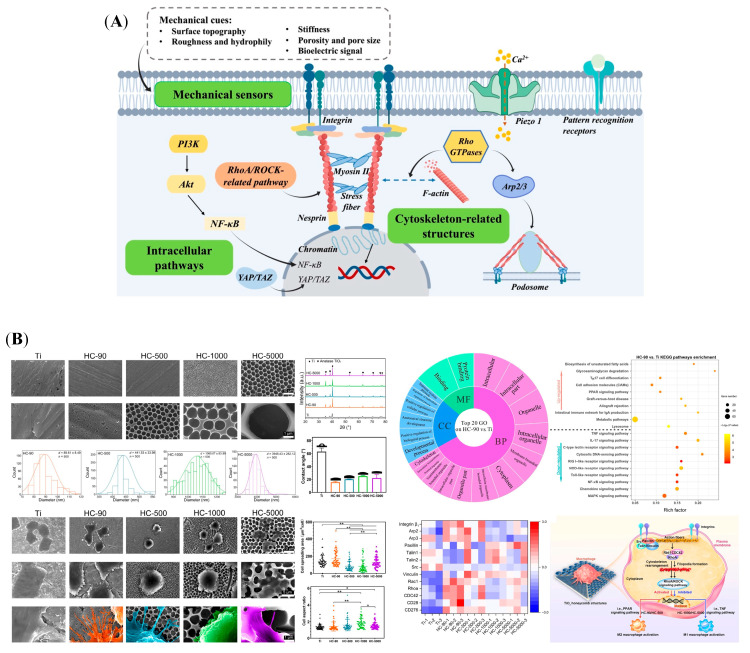
Macrophages transform biophysical cues of biomaterials into biological signals, followed by regulating bone regeneration. (**A**) Potential mechanotransduction pathways for regulating macrophage behavior: mechanical sensors, cytoskeleton-related structures, and intracellular pathways. (**B**) Surface characterizations of TiO_2_ honeycomb-like nanostructure; the morphological changes in macrophages induced by it, and a mechanistic analysis of macrophage polarization. (*: *p* < 0.05; **: *p* < 0.01). Reprinted from Ref. [86].

Living cells in the body are constantly exposed to mechanical stimuli originating from the surrounding microenvironment, which alter cellular fates and regulate their functions [77]. Macrophages, as a type of multi-functional cells, can sense the implanted biomaterials and their surrounding microenvironment [78]. They further transduce these mechanical cues into biological signals, such as surface topography, roughness, hydrophily, porosity, stiffness, and bioelectric signal [79,80]. These transduction ways involve regulating mechanical sensors, rearranging cytoskeleton-related structures, and activating associated signaling pathways (Figure 2A).

Macrophages adhere to the substrates and sense their topography by mechanical sensors, such as integrins and mechanosensitive ion channel proteins [81]. Integrins are transmembrane receptors on the cell surfaces, which assist cell–cell and/or cell-extracellular matrix (ECM) interaction [82]. Lin Lv et al.’s study showed that biomaterial surface hydrophilicity regulated macrophage polarization and osteogenesis through selective expression of integrin β1 or β2, which influenced macrophage behavior [83]. The hydrophilic surface activated the PI3K/Akt pathway to drive M2 polarization by interacting with integrin β1, but the hydrophobic surface resulted in M1 polarization by nuclear factor κ-B (NF-κB) pathway activation, which is associated with integrin β2 [83]. Other study results also suggested that the physical properties of biomaterials modulated macrophage polarization by expressing different integrins, which resulted in the cascade of intracellular pathways, such as PI3K/Akt-NF-κB pathway, c-Jun N-terminal Kinase (JNK) pathway, and RhoA/Rho-associated kinase (ROCK)-related pathway [84,85]. Based on transcriptomic analysis, the Yizhou Zhu et al.’s study revealed that a TiO_2_ honeycomb-like structure also upregulated the RhoA/ROCK signaling pathway to induce M2 macrophage polarization, followed by promoting osteogenic differentiation of MSCs in vitro and facilitating bone-to-implant osteointegration in vivo (Figure 2B) [86]. In addition, the cytoplasmic domains of activated integrin link to actin filaments, by which they transfer extracellular information and rearrange the cell shape [87]. Several cytoskeleton-related proteins and structures participate in this process, such as F-actin, talin, vinculin, and podosomes [88,89]. M1 and M2 macrophages exhibit dramatically different cell shapes, which are mainly controlled by the cytoskeleton [90]. By employing a micropatterning approach to directly control the cytoskeleton structure, the elongation of macrophages induced polarization toward an M2 phenotype [91]. These results implied that actin polymerization, actin/myosin contractility, and ROCK and myosin light chain kinase (MLCK) activities play a crucial role in controlling macrophage polarization by cell shape [91]. Consistently, to precisely design biomaterial porosity, the other investigation cultured human macrophages in poly(ε-caprolactone) (PCL) scaffold with different pore sizes; the results showed that cell elongation tended to increase M2 phenotype polarization by smaller pore size [92]. Further, Vijaykumar S. Meli et al. study showed that substrate stiffness and cytoskeletal polymerization regulated macrophage behaviors by Yes-associated protein (YAP) nuclear localization [79]. These studies suggested that the cytoskeleton dynamics are closely related to macrophage polarization [79]. In addition, proteins in the body adsorb to biomaterial surfaces immediately after implantation, reestablishing an ECM microenvironment [93]. Macrophages can sense the mechanical properties of the ECM through cytoskeleton rearrangement, by which they are polarized toward different phenotypes [82,94]. In addition, mechanosensitive ion channel proteins, such as Piezo1, are also involved in regulating macrophage polarization [95]. Piezo1 plays an important role in sensing the different stiffness of biomaterials and then participating in both inflammatory and healing pathways by mediating calcium ion (Ca^2+^) influx [95]. Above all, the effects of physical cues on macrophages should be considered during the design and development of osteogenic biomaterials.

### 3.2. Biochemical Cues

**Figure 3:**
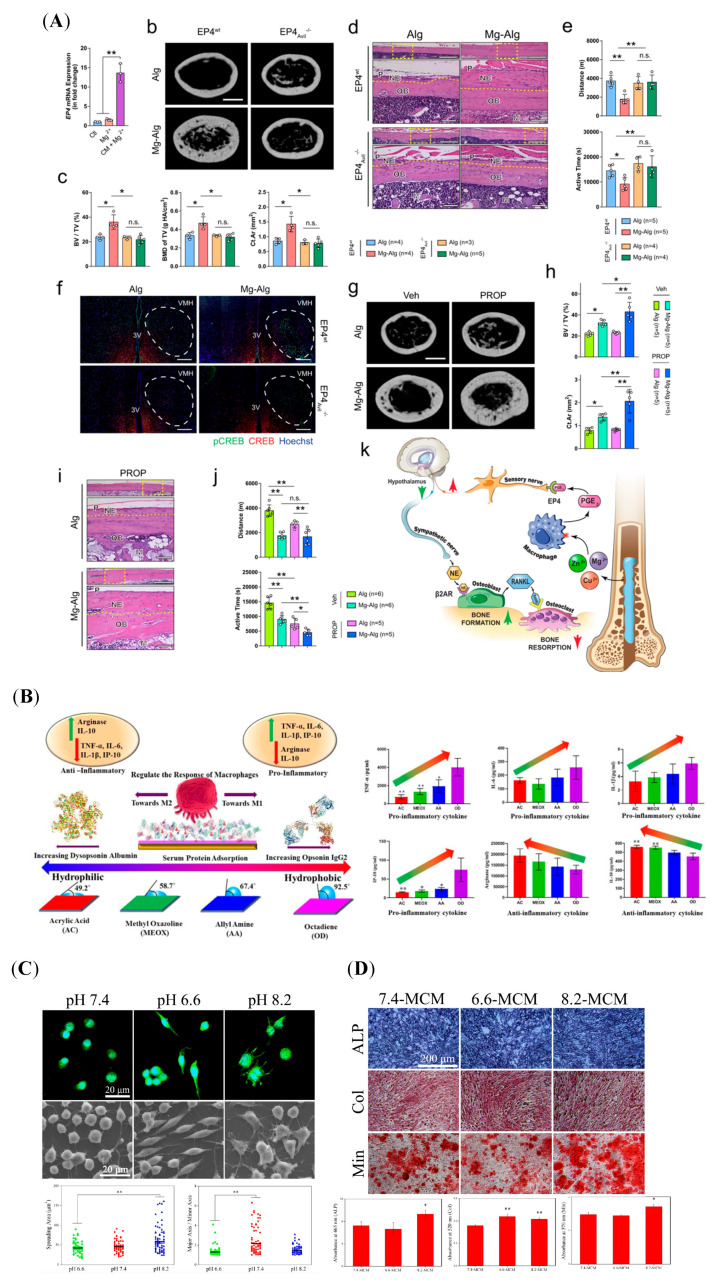
Biomaterials act on macrophages with biochemical signals, followed by regulating bone regeneration. (**A**) The divalent cations increased bone formation and decreased bone resorption by regulating the immune–neural axis. (n.s.: not significant, *: *p*  <  0.05, **: *p*  <  0.01). Reprinted from Ref. [99]. (**B**) The influence of modified-surface wettability in serum protein adsorption and further immune responses in macrophages. (*: *p*  <  0.05, **: *p*  <  0.01). Reprinted with permission from Ref. [16]. Copyright 2019, American Chemical Society. (**C**) The macrophage morphology in response to different pH environments was observed via laser confocal microscopy and scanning electron microscopy (SEM). (Green: FITC-Phalloidin, Blue: 4′-6-diamidino-2-phenylindole (DAPI), **: *p* < 0.01, compared to the pH 7.4 group). Reprinted with permission from Ref. [117]. Copyright 2019, American Chemical Society. (**D**) ALP activity, collagen synthesis, and in vitro mineralization of the OBs cultured by various macrophage-CM for 5, 10, or 14 days, respectively. (*: *p* <0.05, **: *p* < 0.01, compared to the 7.4-MCM group). Reprinted with permission from Ref. [117]. Copyright 2019, American Chemical Society.

Chemical modification methods are widely used to optimize the properties of osteogenic biomaterials. Several chemical cues regulate macrophage polarization, such as ion release, functional groups, and pH, as shown in Figure 3. These factors play crucial roles in modulating the osteoimmune microenvironment by orchestrating inflammatory responses and regulating tissue repair.

Metallic elements are integral to cellular composition and regulate biological behaviors through signaling pathways. Immune cells are sensitive to metal ions released from biomaterials. Ca^2+^ is a key component of bone tissue and plays an immunomodulatory role [96,97]. Controlled Ca^2+^ release from biphasic calcium phosphate (BCP) can activate the calcium-sensing receptor (CaSR) in macrophages, thereby enhancing the expression of M2 macrophage markers via the Wnt/β-catenin pathway [98]. M2-polarized macrophages facilitate the differentiation of MSCs into OBs [98]. Other divalent cations, like magnesium ion (Mg^2+^), zinc ion (Zn^2+^), or copper ion (Cu^2+^), was delivered by the alginate-based hydrogel (Alg), and their underlying mechanisms in promoting bone formation were explored [99]. As shown in Figure 3A, the results of this study demonstrated that the divalent cations triggered prostaglandin E2 (PGE_2_) production from macrophages, which can activate the PGE_2_ receptor4 (EP4) at the sensory nerve to tune down sympathetic tones via the cAMP-response element binding protein (CREB) signaling in the ventromedial hypothalamus (VMH), resulting in the increase in osteogenesis in the periosteum, and decreasing osteoclastogenesis [99]. Zn element loaded onto the TiO_2_ nanotubes’ (TNTs) surface (Zn-incorporated TNT) increased the expressions of M2 markers in macrophages [100]. The conditioned medium of macrophages in the Zn-incorporated TNT can enhance the osteogenic differentiation of OBs [100]. Similarly, R. Zhang et al. reported that micro/nanostructured TiO_2_/ZnO coating produced an immune microenvironment, which promoted osteogenesis [101]. Furthermore, the microarray was used to investigate the gene expression profile of macrophages on Zn-coated sulfonated polyetheretherketone (SPEEK) [102]. The results showed that the M2 markers (CD206 and CD163) and osteogenic genes (BMP-2, VEGF, and TGF-β) were increased in the Zn-coated SPEEK group [102]. In contrast, C-C Chemokine Receptor Type 7 (CCR7), inducible nitric oxide synthase (iNOS), and M1 markers were decreased [102]. The Kyoto Encyclopedia of Genes and Genomes (KEGG) was employed to analyze the pathways [102]. Pro-inflammatory and M1-phenotype-related pathways, TNF and NF-κB pathways, were downregulated, and the pathway regulating M2 polarization and bone formation, Janus kinase (Jak)-signal transducer and activator of transcription (STAT), and VEGF pathways were upregulated [102]. Incorporating more than one element on the biomaterial surface enables the synergistic effect of the elements to be utilized. For example, compared with Ti-Ca and Ti–strontium (Sr), Ti-Ca-Sr was superior in modulating M2 polarization, which increased the osteogenic differentiation of BMSCs in co-culture [103].

The functional groups of biomaterial surfaces play crucial roles in directing immune cell behavior by regulating protein adsorption from serum or reprogramming cell phenotypes. R. M. Visalakshan et al. modified acrylic acid (AC), 2-methyl-2-oxazoline (MEOX), allylamine (AA), and 1,7-octadiene (OD) in the substrate surface by employing plasma polymerization (Figure 3B) [16]. The results showed that protein absorption was influenced by different chemical functionalities, which possessed a range of wettability [16]. Digitally, hydrophilic surface-induced protein absorption increased the release of anti-inflammatory cytokines [104]. In turn, the hydrophobic surface increased the production of pro-inflammatory cytokines [105]. Other studies revealed that modified biomaterials with different functional groups can reprogram macrophage phenotypes, further influencing the tissue microenvironment [106].

The human body usually maintains its pH at a narrow range of 7.35–7.45 [107]. The implanted biomaterials, especially biodegradable bone material, could change the microenvironmental pH around the materials by degrading or releasing ions [108]. Some polymers, like polylactide acid (PLA), polyglycolide acid (PGA), polylactide-co-glycolide acid (PLGA), and PCL, generate several acidic products, subsequently decreasing the surrounding pH [109,110,111,112]. In contrast, bio-ceramic/glass materials can increase the local pH by releasing alkaline ions [113]. Several studies revealed that relatively high-pH environments are more favorable for the synthesis of osteogenesis proteins, such as collagen and ALP [114,115]. In turn, osteoclastic activity was inhibited [116]. In addition, immune cells can alter their phenotypes and cytokine patterns in response to the environmental pH. Hong Wu et al. reported that various environmental pH values induced changes in macrophage morphology and OB mineralization [117], as shown in Figure 3C,D. Specifically, an acidic condition with a pH of 6.6 promotes M2 polarization, evidenced by increasing M2 markers arginase-1 (Arg-1) and CD206 [117]. The alkaline condition with a pH of 8.2 increased the expression of CD11c, TNF-α, IL-1β, and IL-6, indicating an enhancement of M1 polarization [117]. Their results further showed that culturing macrophages in pH 8.2 media increased ATP activity, collagen synthesis, in vitro mineralization, and the expression of ALP, Col-I, and OCN in OBs [117]. Thus, the microenvironmental pH might be an important switch to control macrophage polarization.

### 3.3. Biological Cues

**Figure 4:**
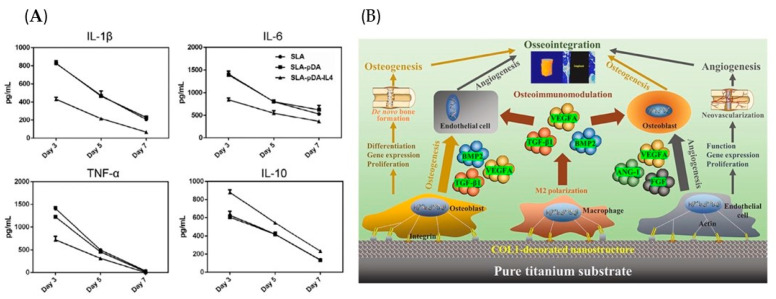
Biomaterials act on macrophages with biological signals, followed by regulating bone regeneration. (**A**) IL-4 loading on the biomaterial surface reduced the release of pro-inflammatory factors and increased anti-inflammatory cytokines from macrophages. Reprinted with permission from Ref. [119]. Copyright 2019, John Wiley and Sons. (**B**) The COL-I decorated nanoporous network on Ti surfaces could significantly regulate early inflammatory reaction and subsequent angio-/osteogenesis processes, resulting in favorable osseointegration. Reprinted with permission from Ref. [127]. Copyright 2022, Elsevier.

Several cytokines and chemokines, such as IL-4, IL-10, BMP-2, and VEGF, regulate the osteoimmune environment. Besides being produced by immune cells responding to biomaterials, they can be released directly from osteogenic biomaterials to act on tissue cells. A study showed that BMP-2 activated macrophages by the pSmad1/5/8 pathway, and the CM harvested from BMP-2-treated macrophages accelerated the osteogenesis [118]. Further, the gelatin sponge incorporated with BMP-2 significantly increased macrophage infiltration and decreased the expression of M1 markers, such as IL-1β, IL-6, and iNOS, simultaneously [118]. In addition, Wang et al. modified the Ti surface with poly(dopamine) (pDA)-assisted immobilization of IL-4 (Figure 4A) [119]. The sandblasted and acid-etched (SLA)-pDA-IL4 surfaces retained IL-4 bioactivity and increased the M2/M1 proportion, which is anticipated to accelerate further bone integration [119].

Bone regeneration is a dynamic and orderly biological process that includes inflammatory reactions, angiogenesis, and osteogenesis [9]. Some cytokines modulate this temporal and spatial pattern to coordinate bone remodeling. Kara et al. designed scaffolds to promote bone regeneration by sequentially switching macrophage polarization [120]. The scaffolds induced M1 polarization by shortly releasing IFN-γ, which plays a role in the early stage of healing [120,121]. Subsequently, they facilitated M2 polarization by releasing IL-4 to promote vascularization [120]. This study suggested that sequential cytokine release may promote angiogenesis by altering M1 and M2 polarization. In addition, a composite scaffold of 3D-printed titanium alloy with BMP-2 and IL-4 could polarize macrophages to the M2 phenotype, resulting in an osteogenic immune microenvironment [122]. Simultaneously, it promoted the differentiation of hBMSCs to OBs [122]. Moreover, BMP-2 exerts osteogenic effects in a dose-dependent manner [123]. High-dose BMP-2 inhibited M1 macrophage infiltration by inducing IL-1Ra secretion from MSCs [124]. Based on the in vitro and in vivo results, the bioactive scaffold accelerated bone regeneration by delivering multifunctional cytokines to elicit synergistic reactions.

Rapid cell adhesion is beneficial for avoiding the encapsulation of non-adhesive proteins after implanting the biomaterials [125]. As a natural polymer, COL-I favors cell initial adhesion by promoting focal adhesion formation [126]. Decorating COL-I on Ti substrates to develop the nanoporous network surfaces (T-ADC) could timely convert macrophages from pro-inflammatory M1 to pro-healing M2 phenotype, creating favorable osteoimmune microenvironments to enhance angio-/osteogenesis (Figure 4B) [127]. Further RNA sequence analysis showed that T-ADC promoted macrophage adhesion, the extension of lamellipodia and filopodia, and M2 polarization by synergistically activating RhoA/ROCK, PI3K-AKT, and classical MAPK signaling pathways [127]. Subsequently, it was proven that T-ADC prominently facilitated the effects of angiogenesis and osteogenesis by culturing cells with the specimen-conditioned medium of macrophages (SCMM), OBs (SCMO), or endothelial cells (SCME) [127]. In vivo experiments consistently revealed that T-ADC generated abundant new bone mass and ameliorative osseointegration [127]. 

**Table 1:** Osteogenic biomaterials were involved in osteoimmunomodulation via biophysical, biochemical and biological cues.

	Engineering Parameters	Properties	Models	Effects	References
Biophysical cues	surface topography	Surface grain size in nano-scale (~100 nm) to micron (~500 nm) range	In vitro: macrophages cultured on the sample surfaces In vivo: bilateral muscle pouches in mice	Nano/micro-topographies of hydroxyapatite (HA) resulted in differential integrin expression in macrophages, subsequently affecting cellular behaviors. The nano-topography could reduce tissue inflammation and promote M2 polarization.	[84]
		Cell micropatterning with 20 μm and 50 μm wide lines.	In vitro: macrophages cultured on the sample surfaces.	Macrophage polarization was regulated by reshaping the actin cytoskeleton. The elongation of macrophages could lead to M2 phenotype marker expression and reduce inflammatory cytokine secretion.	[91]
		Honeycomb-like TiO_2_ structures	In vitro: macrophage-CM In vivo: rat tibia implantation model	Honeycomb-like TiO_2_ structures facilitated macrophage filopodia formation and upregulated the Rho family of guanosine triphosphatases (RhoA, Rac1, and CDC42), which reinforced the polarization of macrophages through the activation of the RhoA/Rho–associated protein kinase signaling pathway.	[86]
		Nano-topography	In vitro: macrophage-CM In vivo: New Zealand white rabbits femoral implantation model	HA nano-particles-Ti formed an osteoimmune microenvironment to promote osteo-/angiogenesis via TGF-β, OPG/RANKL, and VEGF signaling pathways.	[128]
	Roughness	100 nm < Ra < 500 nm	In vitro: macrophage-CM	Following Ra values of Ti surfaces increased, macrophages tended to M1 polarization, followed by the promoting osteoblast differentiation.	[129]
		500 nm < Ra < 2 μm	In vitro: macrophages cultured on the sample surfaces	Only a narrow range of roughness (Ra = 0.51–1.36 μm) in Ti surfaces tended to polarize macrophages toward the M2 phenotype.	[130,131]
			In vitro: macrophage-CM/exosomes In vivo: mouse thigh muscle implantation model	Compared to micron-scale BCP (BCP2, ∼3.07 μm), BCP with submicron-scale structure (BCP1, ∼0.66 μm) facilitated M2 macrophage polarization. BCP1 ceramic markedly elevated miR-142a-5p levels in macrophage-derived exosomes, activating the PTEN/AKT signaling pathway, and consequently guiding the differentiation of MSCs towards osteoblast lineage.	[132]
		Ra > 2 μm	In vitro: macrophages cultured on the sample surfaces.	Following Ra values increased, pro-inflammatory cytokines (TNF-α, IL-6) release increased, and anti-inflammatory cytokines (IL-4, IL-10) release decreased.	[133]
	Hydrophily	hydrophilic	In vitro: macrophage-CM	The hydrophilic micro-rough Ti surfaces switched M1 macrophages to M2 phenotype, and enhanced osteogenesis by reducing inflammation.	[134,135]
			In vitro: macrophage-MSCs transwell co-culture; macrophage-CM; macrophage-T cell direct co-culture In vivo: mouse femoral implantation model	The rough-hydrophilic Ti surface could influence macrophage response to modulate the adaptive immune system, which ultimately controls stem cell recruitment and tissue regeneration.	[78]
			In vitro: macrophages cultured on the sample surfaces. In vivo: mouse femoral implantation model and Csf1r-iCre^+^; Wls^fl/fl^ mice	The Ti surface characteristics of roughness and hydrophilicity regulated macrophage polarization by Wnt signaling, and influenced macrophage to recruit other cells, which is critical to osseous healing.	[136]
		super-hydrophilic	In vitro: macrophages cultured on the sample surfaces	The super-hydrophilic nanotubular surface preferentially activated macrophages toward an anti-inflammatory M2 phenotype under standard conditions, and attenuated M1 responses under LPS stimulation, followed by regulating the microenvironment to accelerate inflammation resolution, facilitate tissue repair, and ultimately promote osseointegration.	[137]
	Porosity and pore size	Nanoporous anodic alumina with 20–200 nm sized pores	In vitro: macrophage-CM	Nanoporous structures regulated macrophage differentiation by changing cellular shape and activating the autophagy pathways. The osteoimmune environment formed by the 50 nm nanoporous structure was beneficial to the osteogenic differentiation of BMSCs.	[138]
		Nanoporous alumina membranes with 20–200 nm sized pores	In vitro: macrophages cultured on sample surfaces In vivo: subcutaneous model in mouse	The 200 nm pores induced a stronger inflammatory response as compared to the alumina with 20 nm pores, which increased macrophage activation in vitro and promoted cell recruitment to generate pro-inflammatory cytokines in vivo.	[139]
		Fibrous scaffolds with box-shaped pores	In vitro: macrophages cultured on the scaffolds	The scaffolds facilitate primary human macrophage elongation accompanied by differentiation towards the M2 type, which was most pronounced for the 40 μm pore size.	[92]
		Scaffold generated from microgel with 40 μm, 70 μm, and 130 μm sizes	In vitro: macrophages were encapsulated in microporous annealed particle scaffolds (MAPS)	The activation levels of M1/M2 macrophages were correlated with changes in morphology, cell motility and nucleus shape regulated by the scaffolds.	[140]
	Stiffness	Polyacrylamide hydrogels	In vitro: macrophages cultured on the sample surfaces In vivo: subcutaneous model in mouse	Piezo1 is a mechanosensor of stiffness in macrophages, and its activity modulates polarization responses.	[95]
		Polyacrylamide gels (PA gels)	In vitro: macrophages cultured on the sample surfaces	Gel stiffness regulated the macrophage behavior by Rho-A kinase (ROCK) and podosome-related pathways, including cell polarization, function and migration.	[141]
	Bioelectric signal	Piezoelectric hydrogel	In vitro: macrophage-CM In vivo: rat large-sized cranial injury model	Cs/Gel/PDA-modified HA/PDAmodified BaTiO_3_ (CG/PHA/PBT) piezoelectric hydrogels activated the PI3K/Akt signaling axis to promote macrophage M2 polarization, followed by accelerating angiogenesis and bone regeneration.	[142,143]
		Ti6Al4V scaffold coated with BaTiO3 (BT/Ti (poled))	In vitro: cyclic loading on macrophage-scaffold composites was applied to form piezoelectric condition; macrophage-CM In vivo: ultrasound stimulation of piezoelectric scaffolds in a subcutaneous rat model; sheep cervical corpectomy model	BT/Ti (poled) facilitated macrophage M2 polarization by inhibiting MAPK/JNK signaling pathway and activating oxidative phosphorylation and ATP synthesis, followed by promoting bone regeneration.	[144]
		β-PVDF film under ultrasound treatment and the release of a localized charge	In vitro: ultrasound was applied as stimulation to cells cultured in the piezoelectric *β*-PVDF film	Ultrasound-stimulated piezoelectric *β*-PVDF film enhanced M1 polarization and inhibited M2 polarization via voltage-gated channels and Ca^2+^-CAMK2A-NF-*κ*B axis to regulate Ca^2+^ influx.	[145]
Biochemical cues	Inorganic ions	Ca^2+^	In vitro: macrophage-CM In vivo: gastrocnemius muscle defect models in mouse lower limbs	BCP-released Ca^2+^ targeted the Wnt/β-catenin signaling pathway and activated Arg1 and IL-10 transcription through the CaSR in macrophages.	[98]
		Ca^2+^ + Sr^2+^	In vitro: macrophages cultured on the sample surfaces	Ca and Sr elements modified the nanoscale topographical Ti surfaces upregulated M2 macrophage phenotype expression.	[146,147]
		Zn^2+^	In vitro: macrophage-CM	Zn released from the Zn-incorporated TiO_2_ nanotube (TNT) biomaterials enhanced gene and protein expression of M2 markers, and M1 markers were inhibited. The macrophage-CM of Zn-TNT group strengthened OB proliferation, adhesion and osteogenic differentiation.	[100]
		Mg^2+^, Zn^2+^, Cu^2+^	In vitro: macrophage-CM In vivo: tunnel defect models in femur; macrophage-depleted mouse model	The divalent cation released from Mg–Alg, Cu–Alg, or Zn–Alg alginate can regulate the calcitonin gene-related polypeptide-α+ nerve fibers by enhancing PGE_2_ secretion from macrophages, followed by downregulating sympathetic activity and promoting new bone formation.	[99]
	Organic functional groups	AC, MEOX, AA), and OD	In vitro: macrophages cultured on the sample surfaces	These organic functional groups regulated the protein adsorption patterns in human serum by forming hydrophilic or hydrophobic surfaces, leading to distinct macrophage polarization.	[16]
	pH	pH6.2–8.6	In vitro: macrophage-CM	The acidic environment (pH 6.6) tended to polarize macrophages to M2 phenotype, while alkaline environment (pH 8.2) led to M1 polarization.	[117]
		PGA scaffold degradation	In vitro: macrophages cultured on the medium with PGA degradation products. In vivo: subcutaneous model in mouse	The fast degradation of porous scaffolds triggered M1 macrophages into the implantation site, whilst the slow degradation of PGA fibers promoted the polarization of macrophages into M2 pro-healing phenotypes.	[110]
Biological cues	Cytokines	IL-10	In vivo: subcutaneous model in rats	IL-10 loaded in the hexamethylenediisocyanate-crosslinked dermal sheep collagen (HDSC) disks downmodulates the foreign body reaction (FBR), impairing the progression of the FBR.	[148]
		IL-4	In vitro: macrophages cultured on the sample surfaces	The SLA-pDA-IL4 surfaces described here are able to activate adherent macrophages into M2 phenotype and reduce the release of pro-inflammatory cytokines.	[119]
	Proteins	BMP-2	In vitro: macrophages- MSCs transwell model In vivo: femoral defect model and subcutaneous model in mice	BMP-2/CPC induced bone regeneration by regulating macrophage-MSC interaction.	[124]
		COL-I	In vitro: macrophage-CM In vivo: Tibia implant model in rats	COL-I decorated nanoporous network on titanium implant surface inhibited inflammation and osteoclastic-related gene expression in macrophages by activating RhoA/ROCK, PI3K-AKT, and classical MAPK signaling pathways, followed by facilitating angiogenesis and osteogenesis.	[127]
spatiotemporal immunomodulation		Phenolic ligand (tannic acid, TA) + indometacin (IND)	In vitro: macrophage-CM In vivo: sample was inserted in the femur of mice	In the normal biological environment, the coating was relatively stable, while TA and IND motifs could be triggered in the inflammatory environment to downregulate pro-inflammatory cytokines and upregulate anti-inflammatory cytokines and osteogenic-related factors.	[149]
		calcium-strontium-zinc-phosphate (CSZP) coating + IL-4	In vitro: BMMSCs and macrophages directly co-cultured In vivo: rat subcutaneous implant model; rat femoral defect repair model	The macrophages were recruited and polarized to M1 by CSZP coating, followed by M2 polarization by adding IL-4.	[150]
		IFN-γ + IL-4	In vitro: macrophages cultured on the sample surfaces In vivo: subcutaneous model in mice	The modified decellularized bone scaffolds shortly released IFN-γ to promote the M1 phenotype, followed by a more sustained release of IL-4 to promote the M2 phenotype.	[120]

## 4. Current Osteoimmunomodulation Models in Evaluating Osteogenic Biomaterials

Osteogenic biomaterial implantation rebuilt a new osteoimmune microenvironment, which mainly involved various bone lineage cells, vascular cells, and immune cells. To enhance a better preclinical evaluation of osteogenic materials, it is necessary to explore further the interaction between biomaterials and surrounding cells from the perspective of osteoimmunomodulation. However, the experimental results usually depend on the model selected [151]. Analyzing the properties of each model can inform the selection of an appropriate model to evaluate the new osteogenic biomaterials. As shown in Table 2, the characteristics of different evaluation models were compared, such as physiological relevance, cost-efficiency, measurement methods, complexity, and translational potential.

### 4.1. Two-Dimensional (2D) Models

The two-dimensional model cultures cells on a flat, two-dimensional surface, such as a culture plate, dish, slide, or transwell, forming a cell layer. These models are widely employed to investigate the cellular crosstalk, signaling pathways, and molecular mechanisms that link the skeletal and immune systems [102,141,145]. In addition, various disease conditions can be mimicked by altering the medium composition. For example, adding high-concentration glucose is used to simulate a diabetic environment, and LPS is added to induce inflammation [152]. While 2D models are cost-effective and facilitate straightforward analysis, they are unable to replicate the three-dimensional osteoimmune microenvironment, thereby limiting their capacity to fully capture complex interactions such as matrix-mediated signaling or spatial relationships within bone tissue [153]. Despite these limitations, 2D models remain a valuable tool for high-throughput screening of biomaterials, drugs, or signaling modulators with the objective of modulating osteoimmune responses.

#### 4.1.1. CM Models

One cell type (donor cell) is first cultured with osteogenic biomaterials in the media to induce biological responses, followed by mixing the media (CM) with the osteogenic medium in a certain proportion, such as 1:1, 1:2, or 1:3 [102,154,155,156]. The CM comprises the components of cytokines, chemokines, and exosomes, which perform various functions and are the essential link between immune and bone systems [157]. The CM model has been widely used in exploring the osteoimmunomodulation characteristics of osteogenic biomaterials, largely due to its simple procedure, convenient storage, straightforward analysis, and no special instrument. Collected CM was employed to culture bone-related cells (acceptor cells), with the objective of evaluating the role of biomaterials-induced immune cells, most notably macrophages, in osteogenesis. In vivo, macrophage polarization adjusts to surrounding stimuli cues, followed by rebuilding the microenvironment by releasing various cytokines and regulating OBs differentiation [158]. Under in vitro models, polarized macrophages release cytokines into the culture media. Thus, the components of CM can represent the polarization condition and be used to explore the interactions between macrophages and bone-related cells. For example, to verify the role of macrophage-CM in OBs, a study investigated the cellular behaviors of BMSCs after culturing in different CMs generated by unpolarized macrophages (M0) or polarized macrophages (M1 or M2) [159]. Based on their results, the M0-CM had a remarkable effect on cell osteogenic differentiation, the M2-CM also facilitated BMSCs osteogenesis, whereas the M1-CM supported the adipogenic differentiation of BMSCs [159]. These findings were consistent with the in vivo results, which means that macrophage-CM contained sufficient components to regulate BMSC differentiation.

**Figure 5:**
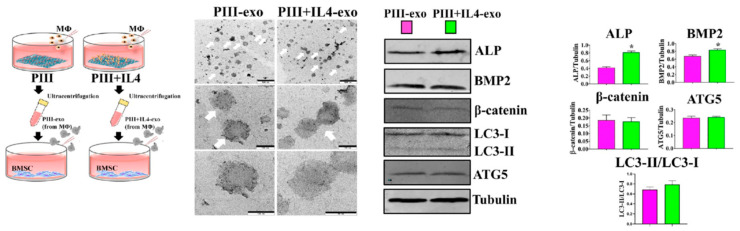
Macrophage-CM works as a medium to transmit biological information between macrophage and bone-related cells. Exosomes (white arrow) were isolated from the media of culturing PIII/PIII + IL4-stimulated macrophages, followed by treating the BMSC (*: *p* <0.05). Reprinted with permission from Ref. [160]. Copyright 2023, American Chemical Society.

The CM is easily accessible, and one or several components can be isolated from culture media, followed by investigating their potential roles in regulating bone formation. For example, as shown in Figure 5, to determine the effects of macrophage exosomes on osteoblast differentiation, exosomes produced by plasma immersion ion implantation (PIII)+IL4-stimulated macrophages were isolated from the supernatants, which can be immediately used in BMSCs culture or stored at −80 °C [160]. The results revealed that PIII + IL4-exosomes significantly increase the expression of osteoblastic differentiation markers [160].

It should be noted that the compositions of the media differ between individual cells, such as glucose concentration, amino acid content, and inorganic ion species [161]. In addition to reacting to the desired cell-secreted factors from donor cells, the acceptor cells can be interfered with by the medium components. These factors have the potential to impact the outcome; however, they can easily be overlooked. In addition, as we mentioned above, there is no standardized ratio of CM (the more frequently used ratios: 1:1, 1:2, or 1:3), which is a crucial factor influencing the cytokine concentration in the CM. The concentration of cytokines secreted by macrophages mediated different effects on the behavior of bone cells [162]. For example, TNF-α, a pro-inflammatory cytokine, showed a concentration-dependent effect on regulating bone homeostasis [163]. Low-level TNF-α stimulates mesenchymal precursor cells to differentiate into OBs, whereas high-level TNF-α is an inhibitor of OB differentiation and an activator of osteoclastogenesis [163]. In practical research, the concentration of CM can be determined by cytotoxicity assays. Different ratios of CM (1:100 to 1) were applied in culturing cells, and 1:1 was chosen for the next experiment based on the results of the MTT assay [164]. Thus, it is better to further optimize the ratio of CM through experiments to establish a standardized model. In addition, the CM model focuses more on the unidirectional effect of macrophages on bone cells by accumulating secreted cytokines in the medium, resulting in ignoring the crosstalk between macrophages and bone cells.

#### 4.1.2. Indirect Co-Culture Models

The indirect co-culture model is characterized by the absence of intercellular physical contact, with cells instead engaging in interactions via paracrine pathways. The transwell insert system has become a standard co-culture system in studies involving two distinct but interacting cell types [165]. To fulfill the requirements of the studies, there are also some models established with three or four cell types [166,167,168].

The transwell system utilizes a semipermeable membrane to separate the cells while enabling the bidirectional exchange of secreted molecules within each well. It consists of an insert with a permeable membrane at its base, placed within a culture dish or well plate to create two chambers: an upper chamber for cell culture and a lower chamber for media or other cell types. The membrane, composed of materials such as polycarbonate or polyester, is available in a 0.4 µm to 8.0 µm range of pore sizes to facilitate selective permeation of substances [169,170], mimicking natural interactions such as nutrient transport or immune cell migration. Due to the dose- and time-dependent effects of inflammatory cytokines on bone regeneration, the transwell system is more suitable for exploring the dynamic impact of osteoimmunomodulation, which can reflect the real-time crosstalk among cells [171]. Fan Zhang et al. designed an ultrasound-controlled hydrogel in situ with self-assembled ultrashort peptide nanofibers (UPN@hydrogel), which can temporally release ultrashort peptide (Ser-Glu-Ser-Ser-Glu, SESSE) to regulate M2-type macrophage polarization, followed by the change in surrounding environment [171]. Based on the released property of this structure, the transwell system was used to further explore the BMSC differentiation, which was influenced by the dynamic immune microenvironment [171]. The transwell is suitable for evaluating the temporally variable osteogenic biomaterials.

**Figure 6:**
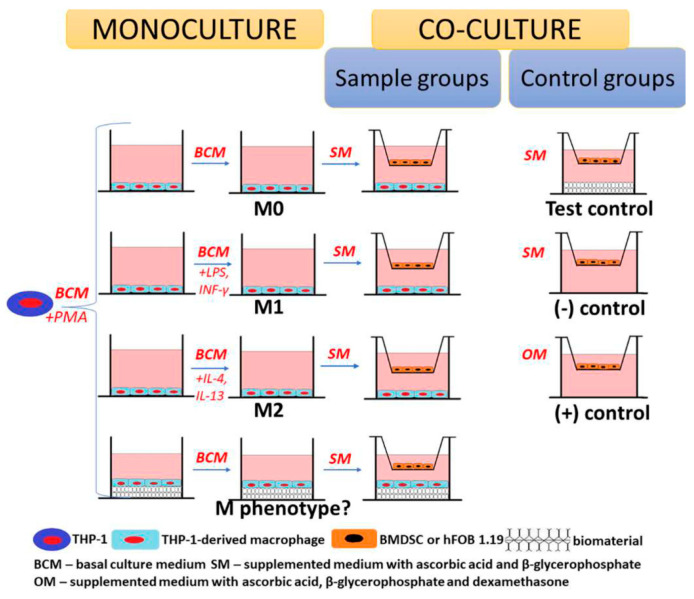
Schematic illustration of the application of the transwell model in cell co-culture. A model of BMSC or OB co-culture with treated macrophages. Chitosan/Agarose/NanoHA bone scaffold-treated macrophages promoted BMSC osteogenic differentiation. Reprinted from Ref. [177].

The transwell system enables compartmentalized cell culture while allowing communication between chambers, making them versatile for modeling in vivo conditions. They are widely employed to investigate cell migration and to construct the barrier models [172,173,174,175]. A study investigated the effects of BCP on BMSC homing by regulating macrophages, in which the transwell system played an essential role [169]. Specifically, macrophages were seeded on the BCP surfaces, followed by collecting the supernatants and cells, which were analyzed with cytokine array and real-time quantitative reverse transcriptase polymerase chain reaction (qRT-PCR), respectively [169]. The results showed that BCP promoted the expression of C-C motif chemokine ligand 2 (CCL2)/MCP-1 and C-C motif chemokine ligand 3 (CCL3)/MIP-1α in macrophages, which were proved to accelerate BMSC migration [169]. The macrophages were further seeded in the bottom of transwell dishes and co-cultured with BMSCs that had been seeded in the inserts [169]. In this system, BMSC migration can be influenced by the cytokines and chemokines secreted by macrophages, and the results suggested that the CCL3/C-C chemokine receptor type 1 (CCR1), CCL2/C-C chemokine receptor type 2 (CCR2) axis may exert a predominant chemotactic effect for BMSC recruitment [169]. Another study functionalized the Ti surface with PTL@Sr coating to enhance osteogenesis and osteoimmunomodulation [176]. In this study, the macrophages were first cultured on the sample surfaces to harvest the CM; the BMSCs were then cultured in macrophage-CM in the transwell system to evaluate the cell migration [176]. Compared with the CM model, the transwell co-culture model allows real-time crosstalk between macrophages, bone cells, and osteogenic biomaterials (Figure 6) [177]. It is more beneficial for exploring the dynamic effect of osteoimmunomodulation. In addition, it can be employed to explore the cell migration and the recruitment of immune cells to bone cells. However, some factors other than experimental variables, like membrane properties and the mixed media, may interfere with the results.

#### 4.1.3. Direct Co-Culture Models

**Figure 7:**
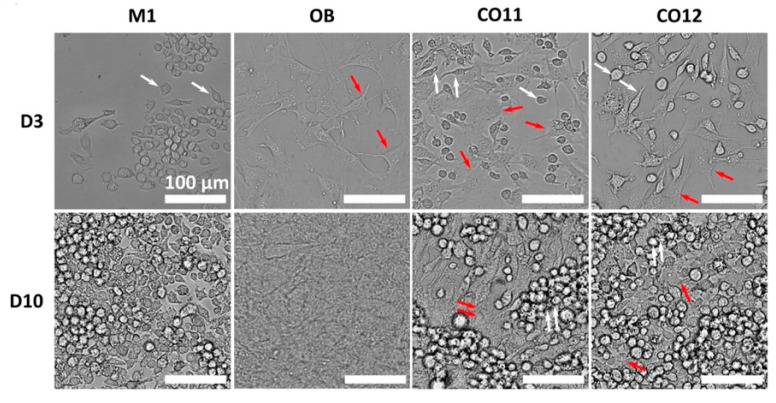
M1 macrophage and OBs direct co-culture model. Spatial distribution of OBs and M1 macrophages after culturing for 3 and 10 days. Reprinted from Ref. [178]. (White arrow: macrophages, red arrow: OBs).

In addition to paracrine pathways, there are complex interrelationships between immune and bone cells, which communicate via cell-to-cell contact. The direct co-culture model means that different cell types are cultured with/in/on the same biomaterial, which is convenient for dynamic communication between cells. Thus, this model can better mimic the in vivo environment by allowing direct contact of cell–cell and/or cell–biomaterials [178]. For example, a study intended to investigate how 3D-printed submicron patterns influence bone regeneration by modulating interactions between OBs and macrophages (Figure 7) [178]. M. Nouri-Goushki et al. established a direct co-culture model to mimic in vivo conditions, allowing the real-time study of cellular interactions. Briefly, they seeded OBs and M1 macrophages in the samples as 1:2 and cultured them with a mixed media of DMEM and α-MEM as 1:1 [178]. The key findings in this study showed that inflammation initially inhibited osteoblast differentiation; however, the patterned surfaces enhanced osteogenic markers like runt-related transcription factor 2 (RUNX2) over time, promoting bone formation [178]. To further reveal the effects of macrophage phenotypic switching on osteogenesis, after co-culturing M1 macrophages and MC3T3-E1 cells, exogenous IL-4 was added at different time points to polarize the M1 macrophage towards M2 [179]. The final results indicated that a transient inflammatory phase is crucial for promoting osteogenesis [179].

Compared with CM and indirect co-culture models, the direct co-culture model involves both cell-to-cell/biomaterials contact and soluble factor interactions. The effects of these three models on pro-osteogenesis were evaluated in a study [151]. The results suggested that the capacity of macrophages to improve osteogenic differentiation and mineralization was as follows: direct co-culture model > indirect co-culture model > CM [151]. Therefore, a more appropriate experimental model should be chosen according to the purpose of the experiment.

The direct co-culture model also presents several challenges and limitations. One significant disadvantage is the complexity of this system, which can make it difficult to distinguish the specific contributions and mechanisms of direct cell–cell contact from those of soluble factors. Additionally, a direct co-culture system is susceptible to the ratios and densities of the different cell types. It is, therefore, essential to optimize the parameters through adequate pre-experiments to avoid dominance by one cell type. Moreover, the dynamic and interconnected signaling processes place a greater demand on testing tools. In some cases, it was necessary to isolate the cells prior to conducting the assay. In addition, similarly to another co-culture model, it should be noted that the culture media is also an important factor in influencing the results [165].

### 4.2. Three-Dimensional Models

Traditional 2D culture models are widely used in studies due to their simplicity and convenience. However, in these models, the cells are mostly in a flattened monolayer growth pattern, which limits the accumulation of ECM and the multidirectional cell-to-cell interactions [180]. To evaluate the osteogenic biomaterials in conditions closer to the in vivo environment, 3D models are increasingly being used in studies [181]. In the dynamic osteogenesis process, the secretion and mineralization of ECM play crucial roles, which can be influenced by the interactions between immune and bone cells. The two most commonly used 3D models for osteoimmunomodulation of osteogenic biomaterials are described below.

#### 4.2.1. Three-Dimensional Scaffold Models

To restore and replace damaged bone tissue, the concept of bone tissue engineering (BTE) was introduced, and its design is undergoing continuous optimization [182]. The BTE typically involves three main components: cells, scaffolds, and bioactive molecules. Scaffold, as the framework, provides mechanical support and 3D structure for cells to adhere, proliferate, and differentiate. The ideal scaffold is usually designed to mimic the physical and chemical properties of natural bone, including porosity, mechanical strength, and biodegradability [183]. Recently, osteoimmunomodulation has also been known as an important indicator for evaluating biomaterials. Min He et al. designed a tannic acid–indometacin (TA-IND) nanoparticle-loaded PCL scaffold, which can spatiotemporally manipulate the osteoimmune microenvironment by scaffold biodegradation and releasing drugs [184]. In this study, the LPS-treated macrophages (M1) were seeded in the nanofibrous scaffold, which was converted to an anti-inflammatory M2 type by the releasing drugs from scaffolds [184]. However, this early process was slow and controlled by the “shielding effect” of the substrate to avoid over-inhibiting the activation of M1 macrophage, which is beneficial to tissue regeneration [185]. At the later stage, the collapse of the scaffolds exposed a greater quantity of TA-IND, effectively relieving the biodegradation-induced chronic inflammation [184]. This 3D model was able to control drug delivery and enabled the facilitation of macrophage polarization [184]. In addition, the scaffold model can provide a 3D space for direct co-culture of multiple cells, thus better simulating the cell–cell contact. The scaffolds synthesized by COL-I were used in a study, and macrophages and OBs were encapsulated in the 3D scaffolds or cultured on the 2D surface [186]. The results of culturing OBs indicated that the scaffold was beneficial in forming the mineralized matrix and upregulated the NF-κB expression, compared to the results of 2D culturing [186]. The macrophages and OBs were further co-encapsulated in the scaffolds to explore their interactions [186]. This 3D collagen matrix provided a closer physical environment, which may account for the difference in results from the 2D model [186]. Similarly, other studies also showed the different behaviors of macrophages and OBs in the 3D model and 2D model [187,188].

Despite significant advancements, 3D scaffolds still have some limitations. It is difficult to completely control the material biodegradation, which can be influenced by its inherent components, design, and the surrounding complex microenvironment. Uneven degradation rates may cause mechanical instability and disrupt tissue regeneration. At the same time, some degradation byproducts can disrupt the microenvironmental homeostasis, such as influencing the surrounding pH value, inducing inflammatory response, or cytotoxicity. Therefore, further development of scaffold models is required to more accurately simulate in vivo physiological conditions.

#### 4.2.2. Microfluidic Platforms

**Figure 8:**
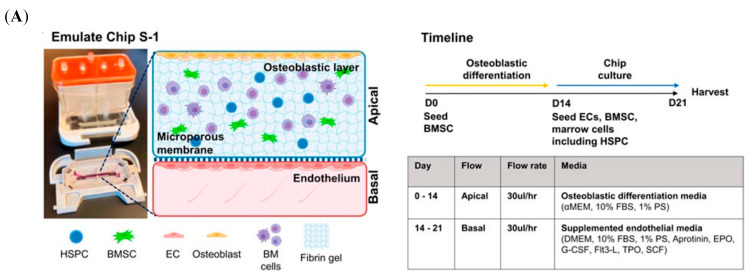

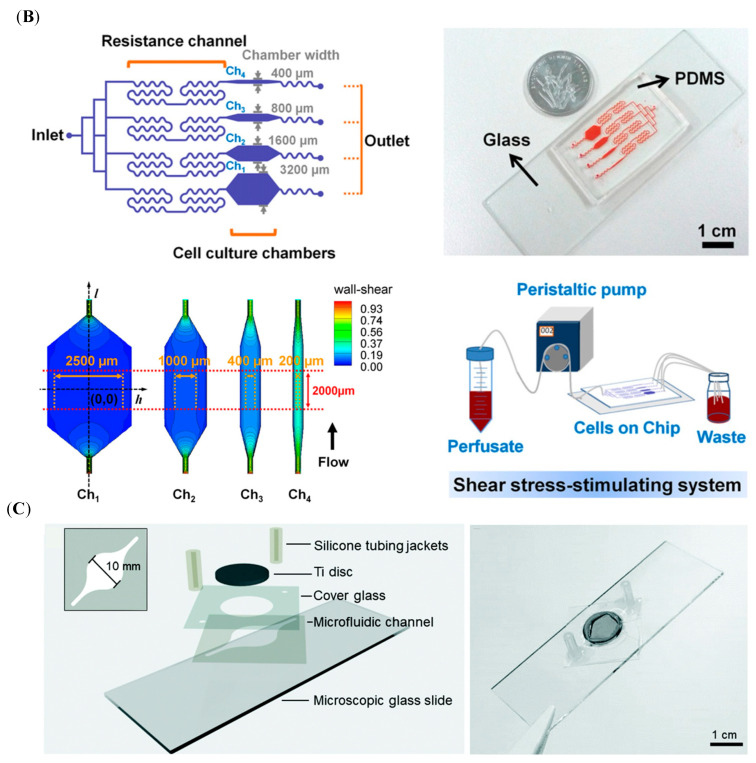
Schematic illustration of the microfluidic models. (**A**) BMME-on-chip with osteoblastic, endothelium layer and whole bone marrow cells, as well as the culture timeline. Reprinted from Ref. [189]. (**B**) A schematic illustration of the microfluidic shear device design and shear stress-stimulating system. Reprinted with permission from Ref. [194]. Copyright 2011, Elsevier. (**C**) The different layers of the Ti-glass-chip and a top-view photograph of the completed device. Reprinted from Ref. [197].

With the development of research technologies and methodologies, the microfluidic system has been used in the field of bone research (Figure 8A) [189]. To establish the interactional models of bone and immune systems, various microfluidic devices have been designed, such as conditional culture by CM, co-culture of immune and bone cells [190], purpose-dependent structures, and multi-organ on one chip.

Different from the microenvironment provided by the traditional model, the microfluid platform is a more dynamic and tunable system, characterized by the bionic mechanical environment. The OB behaviors, such as proliferation, differentiation, and mineralization, are sensitive to mechanical stimuli from the surrounding environment [191,192]. The results of culturing the MSCs and adipose-derived stem cells under the same mechanical stimulation in a microfluidic device show that MSCs were more sensitive and had a higher potential in osteogenic differentiation [193]. As shown in Figure 8B, a multi-shear microfluidic system was developed to measure Ca^2+^ concentration dynamics of osteoblast cytosolic, which can provide four-level shear flows [194]. After treatment with the different shear flows, the OBs cultured in separate chambers showed different Ca^2+^ concentrations in the cytosolic [194]. These results suggested that the in vitro models without the mechanical environment may influence the study outcomes [194]. Another study was further consistent with these results. A 3D osteocyte network was reconstructed by assembling murine early osteocytes (MLO-A5) in the BCP microbeads [195]. After culturing for three weeks, the expression trend of Sost gene in MLO-A5 cells cultured in microfluidic chambers exhibited consistency with that observed in vivo, which is a crucial osteocyte-specific marker for the mechanotransduction function [195]. However, cells cultured in a 2D model did not exhibit a similar trend [195]. Similarly, these mechanical stimuli can modulate immune cell functions. For example, leukocytes tended to migrate toward areas of higher shear stress, which is a crucial process in immune response [196]. Thus, the shear stress of dynamic fluid flow should be optimized to construct an osteoimmune microfluidic model with a closer physiological environment.

The construction of a microfluidic platform of the osteoimmune model offers a novel approach to assessing osteogenic biomaterials. To evaluate the biological properties of medical grade Ti disks for bone repair, Sarah-Sophia D. Carter et al. fabricated two different microfluidic channel designs, Ti-polydimethylsiloxane (PDMS)-chip and Ti-glass-chip [197]. Based on this study, considering the inertness of the materials, the robustness of the fabrication processes, the accessibility of the equipment, and the integration with standard biochemical assays, the Ti-glass-chip was better suited for longer-term cell experiments [197]. The schematic illustration of Ti-glass-chip is shown in Figure 8C [197]. In addition, the microfluidic system can mimic intricate physiological and pathological conditions and evaluate the whole-body level through the real-time observation of communication between different unions. It could link the engineered tissues, such as bone, immune, and vasculature, via vascular flow containing circulating monocytes [198]. This allowed for maintaining dynamic interactions among tissues and the surrounding environment [199], including the osteoimmune microenvironment. Thus, the establishment of a microfluidic platform is a reasonable methodology to mimic the immune environment of bone tissue to evaluate biomaterials.

It is convenient to investigate the dynamic interactions of cell–cell, cell–biomaterials, and cell–microenvironment in the microfluidic platform because it allows the co-culture of multiple cell types and biomaterials. In addition, microfluidic devices can integrate multiple experimental conditions on a single platform, enabling high-throughput screening and real-time monitoring of various cellular responses [200,201,202]. However, there are still some limitations of microfluidic platforms in evaluating osteogenic biomaterials. The adaptation of standard biological assays and imaging techniques to microfluidic systems is also a challenging process, requiring specialized optimization. Moreover, operating these devices demands technical expertise, and issues such as clogging or leakage can impact reproducibility. These factors, combined with cost and accessibility concerns, make microfluidic platforms less universally applicable despite their precision and innovative potential.

#### 4.2.3. Bone Organoids

Bone organoid is a 3D in vitro cell culture system that provides a more comprehensive view of the interactions and spatial patterns between multiple cells, and cells and matrices [203]. It closely mimics the structural and functional microenvironment of native bone tissue, involving a series of highly coordinated biological events, such as cellular self-organization, osteogenic differentiation, ECM secretion, and mineralized collagen formation [204,205]. This cascade of developmental processes relies on complex intrinsic regulatory mechanisms, which cannot be accomplished by traditional 2D or 3D models. Mengru Zhu et al. developed a dynamic DNA/Gelatin methacryloyl (GelMA) hydrogel (CGDE) to recapitulate key biochemical and mechanical features of the bone ECM, providing a supportive microenvironment for bone organoid formation [205]. This dual-network hydrogel facilitated cellular migration, enhanced cellular self-organization, and downregulated innate immune responses, promoting woven bone organoid (WBO) formation via intramembranous ossification [205]. In addition to simulating the physiology of bone tissue, it is useful for exploring disease processes. A trabecular bone organoid model was developed to reproduce the bone remodeling cycle and its spatiotemporal profiles, which can be used to address issues such as osteoporosis and reduced bone density [206]. Thus, it depends on the bone organoid status and properties to apply them in mimicking bone diseases, promoting bone regeneration, and screening biomaterials [207,208]. Based on the highly bionic nature of bone organoids, they will be the next generation of in vitro models for evaluating osteogenic biomaterials and have great potential for clinical translation. However, to further acquire reliable safety, it should be considered in enhancing organoid matrix biocompatibility, precisely controlling cellular behavior, and standardizing fabrication techniques [205].

### 4.3. In Vivo Models

In vitro experiments play a pivotal role in evaluating the direct cell response to osteogenic biomaterials and exploring the potential mechanisms. The animal models represent a crucial intermediate step between in vitro studies and clinical trials, offering comprehensive assessments, such as biocompatibility, mechanical stability, and safety [209]. They provide a realistic and complex in vivo environment that reflects the interactions between the immune system and bone remodeling processes [210]. These models make it possible to observe how biomaterials influence immune responses, such as immune cell activation and cytokine collapse, and how these, in turn, impact bone formation [211]. In addition, animal models can simulate bone defects in different physiologic microenvironments of the human body. To evaluate the bone regeneration biomaterials applied in the alveolar bone, an animal alveolar defect model is beneficial to reflect the distribution the forces from chewing, which may disturb the bone regeneration process [212]. For loss of tooth-supporting tissues due to periodontitis, inhibiting the colonization of periodontal pathogens is an effective strategy to promote the recovery of periodontal hard tissues [213]. Modeling periodontal inflammation can better respond to the interaction of biomaterials with oral microorganisms and their role in regulating immune responses and osteogenesis in the oral complex environment [214]. In addition, animal models can more accurately reflect the degradation of biomaterials because it is difficult to completely simulate complex tissue microenvironments in vitro [110]. Compared to cellular experiments in vitro, animal models also enable long-term observation of potential chronic immune reactions, material degradation, or inflammation, which could interfere with bone regeneration and provide evidence on biocompatibility and safety before clinical trials.

**Figure 9:**
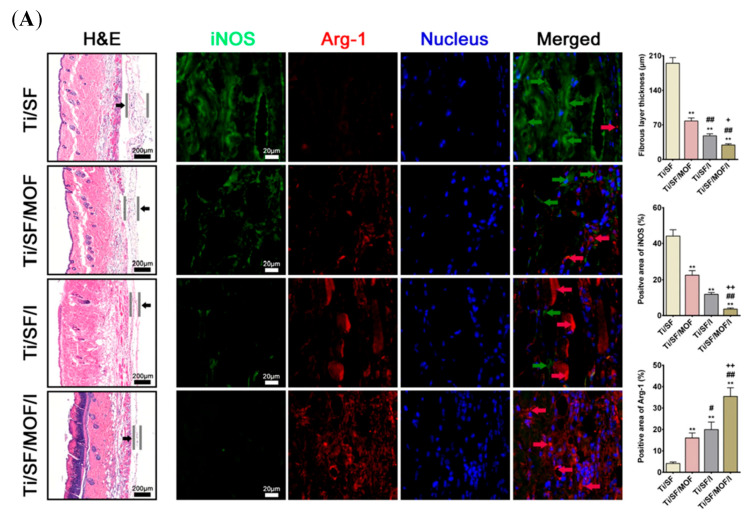

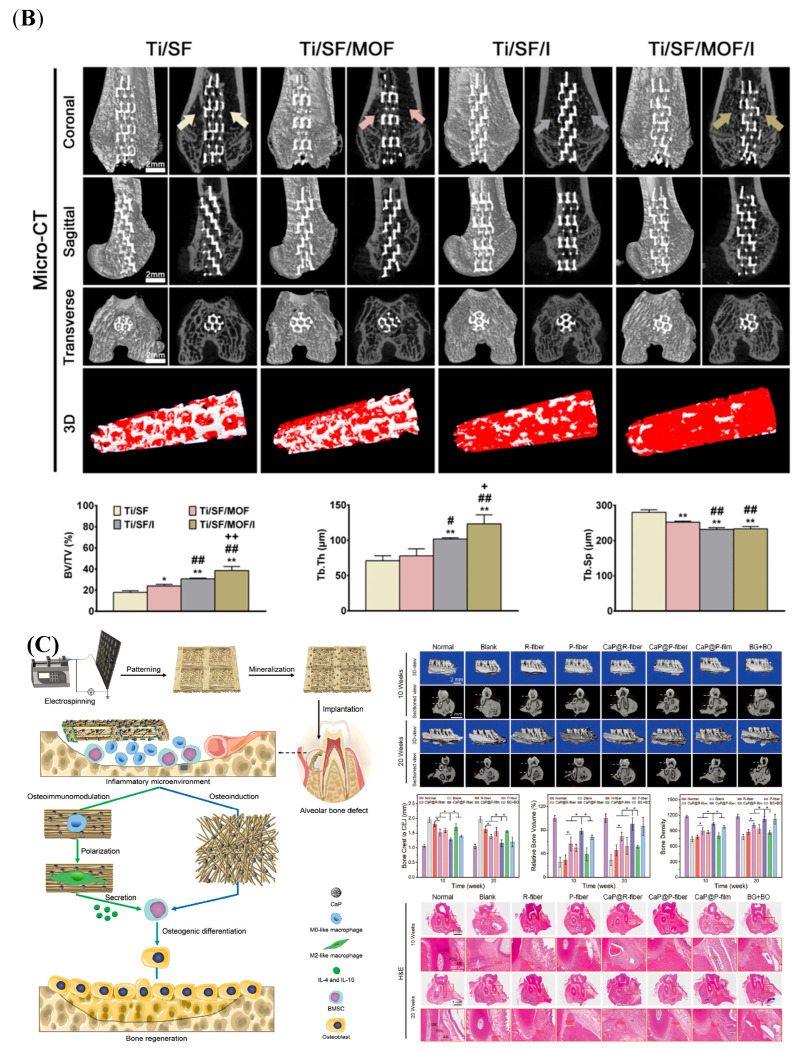
The application of animal models in osteoimmunomodulation evaluation. (**A**) In vivo evaluation of the skin in air pouch model. (# and + represent *p*  <  0.05 when compared with Ti/SF, Ti/SF/MOF, and Ti/SF/I, respectively; **, ##, and ++ represent *p*  <  0.01). Reprinted from Ref. [155]. (**B**) Micro-CT analysis of osteoporotic osseointegration. (*, # and + represent *p*  <  0.05 when compared with Ti/SF, Ti/SF/MOF, and Ti/SF/I, respectively; **, ## and ++ represent *p*  <  0.01). Reprinted from Ref. [155]. (**C**) Schematic illustration of hierarchical-structured mineralized nanofiber (HMF) scaffold for enhancing alveolar bone repair (**Left**). In vivo bone regeneration assessments, including micro-CT evaluation and H&E staining (**Right**) (* *p* < 0.05). Reprinted with permission from Ref. [220]. Copyright 2021, John Wiley and Sons.

Depending on the purpose of the experiment, different animal models are established in the studies. In addition to assessing the osteogenic capacity, the osteoimmunomodulation model also focused on the osteoimmune microenvironment and explored the intrinsic connection between the bone and immune systems. The air pouch model is commonly employed to evaluate the biocompatibility and immunomodulatory effects of biomaterials [215]. Compared to implanting in hard tissue, biomaterial samples are easily inserted into the subcutaneous tissue at the animal back [95,110,120,124]. This model is commonly constructed in mice or rats without the need for large animals. In addition, the exudates and peri-implant tissue can be collected for a comprehensive analysis, such as inflammatory cytokines, infiltration of immune cells, and fibrous encapsulation [216]. A study established a mouse air pouch model and injected carrageenan solution into the pouch [216]. The cells were harvested from the pouch and showed that a significant cell infiltration occurred in response to carrageenan, and the cell types changed over time [216]. In addition, a subcutaneous air pocket was developed on the back of mice by injecting the sterile air, followed by insertion into the scaffold materials [217]. Four days later, the skin tissues were harvested and evaluated [155]. As shown in Figure 9A, the thinnest fibrous layer was observed in the skin sections of modified samples [155]. The immunofluorescence staining further confirmed that this fibrous layer had more Arg-1 expression and less iNOS expression [155]. The bone integration model was also established in this study. A rat femur defect was created first, and the cylindrical implant was then inserted parallel to the long axis of the femur [155]. To observe the new bone formation, the Micro-CT was employed to scan the femur samples containing the implants, and the images and data were quantitatively analyzed by the software (Figure 9B) [155]. Besides the femur defect model [218], depending on the composition and size of the osteogenic material and the implantation site in the human body, other models were constructed in the studies, such as the tibia defect model [219], alveolar bone defect model (Figure 9C) [220], and calvarial bone defect model [221]. Analyzing the data from the air pouch model and bone integration model together facilitates a more comprehensive analysis of osteogenic mechanisms in biomaterials. Taken together, animal models offer crucial insights into how biomaterials interact with both bone and immune systems, guiding their development for clinical applications. However, it is essential to acknowledge the financial, ethical, and individual variability inherent to animal models as a research tool.

## 5. Future Outlook

Based on the above theoretical and practical foundations, we recommend that the osteoimmunomodulation evaluation of osteogenic biomaterials can be used to complement the ISO 10993 guideline [222]. Incorporating this characteristic into guidelines for the evaluation of implants can help prevent implant failure due to osteoimmune microenvironment plays a central regulatory role in the bone regeneration process. Immune cells (e.g., macrophages and T cells) directly regulate osteogenic differentiation of MSCs and osteoclast activity by secreting pro-inflammatory or anti-inflammatory factors, and the early immune response after biomaterial implantation significantly affects subsequent osseointegration results. Traditional evaluation systems focus only on the mechanical properties and in vitro osteogenic capacity of biomaterials, but ignore the decisive role of immune response on bone regeneration, which may lead to biomaterial failure in vivo due to excessive inflammation or immune rejection. By complementing osteoimmune indicators such as macrophage polarization, cytokine dynamics, and immune cell infiltration and phenotyping, it can accurately predict the long-term performance of biomaterials. In particular, the development of microfluidic chips and bone organoid technologies will be beneficial in guiding the design of osteogenic biomaterials with immunomodulation functions, followed by solving the clinical challenges such as osteoporosis and infected bone defects. This integrated strategy will promote the paradigm shift in biomaterials from “passive osteogenesis” to “active immune-osteogenic co-regulation” and significantly increase the success rate of clinical translation.

## 6. Conclusions

The bone and the immune systems are closely linked in the tissue origin and functioning. There is growing evidence that the success of osteogenic biomaterials depends on the osteoimmune microenvironment in which they facilitate. In the early stage of biomaterial implantation, bone damage triggers the acute inflammatory response. The immune cells play an anabolic role in eliminating tissue debris and dead cells while recruiting bone precursor cells through releasing cytokines. In the later phase of bone integration, long-term chronic inflammation produces a catabolic effect that induces failure of bone formation. The osteogenic biomaterials communicate with the surrounding microenvironment, including immune and bone-related cells, via biophysical cues, biochemical cues, and biological cues, resulting in the immune cell conversion from the pro-inflammatory phenotype to the pro-restorative phenotype, followed by the reconstruction of the osteoimmune microenvironment. Understanding the complex spatiotemporal interactions among osteogenic biomaterials, immune cells, and bone-related cells can help guide the biomaterial design and drive advances in BTE.

The development and application of osteogenic biomaterials require rational experimental models for evaluation. In this review, we introduced the current osteoimmunomodulation models for evaluating osteogenic biomaterials and discussed their properties. The CM is the most common experimental approach because it is easy to operate and does not require a special instrument. However, it overlooks the dynamic interactions between cell-to-cell and cell-to-biomaterials. To address this limitation, indirect and direct co-culture models provide a communication platform for cells and biomaterials, enabling their real-time interaction. Besides these traditional approaches, to better mimic the spatial and temporal variability of the osteogenic process in vivo, 3D scaffolds, microfluidic systems, and bone organoids were used to evaluate osteogenic biomaterials. In particular, microfluidic platforms and bone organoids, which can combine bone and the immune system in one system, are used to evaluate osteogenic biomaterials from the organoid level. Although animal experiments remain indispensable in the current evaluation of the osteoimmunomodulatory effects of osteogenic biomaterials, it is promising to build a bone-immuno-biomaterials assay platform with the advancement of microfluidic and single-cell technologies, which can realize the integration of cell culture-measure-analysis and partially replace animal models. Taken together, this paper is beneficial in further standardizing experimental protocols and facilitating the optimization of osteogenic biomaterials by reviewing the current applications of osteoimmunomodulation models in evaluating osteogenic biomaterials.

## Figures and Tables

**Table 2:** Comparison of different assessment models.

Models		Physiological Relevance	Cost-Efficiency	Measurement Methods	Complexity	Translational Potential
2D models	CM models	Paracrine effects	+	1. Easy to separate cell types for measuring RNA/protein expression and staining. 2. Separate evaluation of cell supernatant composition.	+	1. Providing theoretical and mechanistic evidence. 2. Limitations in the presentation of spatiotemporal characteristics of cellular phenotypes and states.
	Indirect co-culture models	Real-time paracrine effects	++	1. Can separate cell types for measuring RNA/protein expression. 2. Evaluation of cell migration.	++	
	Direct co-culture models	Real-time paracrine effect and contact-dependent behaviors	++	Assaying separately for RNA/protein expression after sorting cells.	+++	
3D models	3D scaffold models	Natural bone-like structures	+++	1. Observation of cells growing on the scaffold after staining. 2. Collecting cells by enzymatic digestion of scaffold biomaterials or PBS rinsing, followed by characterizing. 3. Measuring RNA/protein expression after direct lysis of the entire scaffold-cell samples	++++	1. Bridging the gap between 2D cultures and animal models, in particular, bone organoids provide a human-relevant platform. 2. The construction of a platform for simulating tissue homeostasis needs to be further developed.
	Microfluidic platforms	Dynamic bionic mechanical environment	++++	Real-time analysis cell conditions, including scRNA-seq, live-cell imaging, and impedance-based viability monitoring	+++++	
	Bone organoids	Structural and functional microenvironment of native bone	+++++	1. Temporal evaluation by live-cell imaging to track cells in real-time and time-lapse micro-CT to visualize mineralization kinetics. 2. Spatial variability characterized by scRNA-seq and spatial transcriptomics to evaluate gene expression heterogeneity across zones	++++++	
In vivo models		Realistic and complex in vivo environment	++++++	Histological staining and Micro-CT analysis	+++++++	1. Necessary safeguards prior to clinical trials. 2. Limitations in species-specific.

A higher number of + signs indicates that the characteristic is more significant.

## Data Availability

No new data were created or analyzed in this study. Data sharing is not applicable to this article.

## Abbreviations

The following abbreviations are used in this manuscript

AA	Allylamine
AC	Acrylic acid
Akt	Protein Kinase B
Alg	Alginate-based hydrogel
ALP	Alkaline phosphatase
APCs	Antigen-presenting cells;
Arg-1	Arginase-1
BCP	Biphasic calcium phosphate
BMP-2	Bone morphogenetic protein 2
BMP-6	Bone morphogenetic protein 6
BMSCs	Bone marrow mesenchymal stem cells
BTE	Bone tissue engineering
CaSR	Calcium-sensing receptor
CCL2	C-C motif chemokine ligand 2
CCL3	C-C motif chemokine ligand 3
CCR1	C-C chemokine receptor type 1
CCR2	C-C chemokine receptor type 2
CCR7	C-C Chemokine Receptor Type 7
CM	Conditioned media
COL-I	Type I collagen
CREB	cAMP-response element binding protein
CyTOF	Cytometry by Time of Flight
Cu2+	Copper ion
DAMPs	Danger-associated molecular patterns
DAPI	4′-6-diamidino-2-phenylindole
DCs	Dendritic cells
ECM	Extracellular matrix
EP4	PGE2 receptor4
EVs	Vesicles
FBR	Foreign body response
FGF-2	Fibroblast growth factor-2
HOBs	Human osteoblasts
HUVECs	Human umbilical vein endothelial cells
MEOX	2-methyl-2-oxazoline
Mg2+	Magnesium ion
MLCK	Myosin light chain kinase
MMP-9	Matrix metalloproteinase-9
MIP-1	Macrophage inflammatory protein-1
iNOS	Inducible nitric oxide synthase
IFN-γ	Interferon-γ
IL-1β	Interleukin-1β
IL-4	Interleukin-4
IL-6	Interleukin-6
IL-10	Interleukin-10
IL-13	Interleukin-13
IL-17	Interleukin-17
IL-22	Interleukin-22
IL-26	Interleukin-26
JAK	Janus kinase
JNK	c-Jun N-terminal kinase
KEGG	Kyoto Encyclopedia of Genes and Genomes
LTB4	Leukotriene B4
MCP-1	Macrophage chemoattractant protein-1
OBs	Osteoblasts
OCN	Osteocalcin
OCs	Osteoclasts
OD	1,7-octadiene
OPN	Osteopontin
OPG	Osteoprotegerin
OSM	Oncostatin M
OVX	Ovariectomy
PCL	Poly(ε-caprolactone)
PDMS	Polydimethylsiloxane
PD-1	Programmed cell death-1
pDA	Poly(dopamine)
PD-L1	PD-ligand 1
PGA	Polyglycolide acid
PGE2	Prostaglandin E2
PI3K	Phosphoinositide 3-kinase
PIII	Plasma immersion ion implantation
PLA	Polylactide acid
PLGA	Polylactide-co-glycolide acid
pre-B	Precursors of B
qRT-PCR	Real-time quantitative reverse transcriptase polymerase chain reaction
RANKL	Receptor activator of nuclear factor κ-B ligand
ROS	Reactive oxygen species
RUNX2	Runt-related transcription factor 2
SCME	Specimen-conditioned medium of endothelial cells
SCMM	Specimen-conditioned medium of macrophages
SCMO	Specimen-conditioned medium of osteoblasts
SDF-1	Stromal cell-derived factor 1
SDF-1α	Stromal cell-derived factor-1α
SEM	Scanning electron microscopy
SLA	Sandblasted and acid-etched
SPEEK	Sulfonated polyetheretherketone
Sr	Strontium
STAT	Signal transducer and activator of transcription
TGF-β	Transforming growth factor-β
Th cells	Helper T cells
Ti	Titanium
TNF-α	Tumor necrosis factor α
TNTs	TiO_2_ nanotubes
T-ADC	Decorating COL-I on Ti substrates to develop the nanoporous network surfaces
2D	Two-dimensional
VEGF-A	Vascular endothelial growth factor A
VMH	Ventromedial hypothalamus
YAP	Yes-associated protein
Zn^2+^	Zinc ion
